# Taxonomic bias in biodiversity data and societal preferences

**DOI:** 10.1038/s41598-017-09084-6

**Published:** 2017-08-22

**Authors:** Julien Troudet, Philippe Grandcolas, Amandine Blin, Régine Vignes-Lebbe, Frédéric Legendre

**Affiliations:** 1 Institut de Systématique, Evolution, Biodiversité, ISYEB – UMR 7205 MNHN CNRS UPMC EPHE, Sorbonne Universités, 45 rue Buffon, 75005 Paris, France; 2 Outils et Méthodes de la Systématique Intégrative, OMSI – UMS 2700 CNRS MNHN, Muséum national d’Histoire naturelle, CP26, 57 rue Cuvier, 75231 Paris Cedex 05, France

## Abstract

Studying and protecting each and every living species on Earth is a major challenge of the 21^st^ century. Yet, most species remain unknown or unstudied, while others attract most of the public, scientific and government attention. Although known to be detrimental, this taxonomic bias continues to be pervasive in the scientific literature, but is still poorly studied and understood. Here, we used 626 million occurrences from the Global Biodiversity Information Facility (GBIF), the biggest biodiversity data portal, to characterize the taxonomic bias in biodiversity data. We also investigated how societal preferences and taxonomic research relate to biodiversity data gathering. For each species belonging to 24 taxonomic classes, we used the number of publications from Web of Science and the number of web pages from Bing searches to approximate research activity and societal preferences. Our results show that societal preferences, rather than research activity, strongly correlate with taxonomic bias, which lead us to assert that scientists should advertise less charismatic species and develop societal initiatives (e.g. citizen science) that specifically target neglected organisms. Ensuring that biodiversity is representatively sampled while this is still possible is an urgent prerequisite for achieving efficient conservation plans and a global understanding of our surrounding environment.

## Introduction

Since the first Convention on Biological Diversity in 1992, biodiversity and the consequences of its destruction have become a central concern for biologists^1–3^. From scientists to the lay public or policy makers and practitioners, the need to study and protect biodiversity is growing, and scientists have shown that it must be tackled at the gene, species and ecosystem level^4^. Within a context of global change and accelerated biodiversity loss, this necessity has become a major concern and challenge for the 21^st^ century^5, 6^. However, discussions on biodiversity often only focus on a small subset of species, while most of the eukaryotic biodiversity remains unknown or ignored^7, 8^.

Taxonomic bias, also referred to as taxonomic chauvinism^9^, is pervasive in biodiversity research. This bias stems from disparities in our knowledge of different organisms, and in the extent to which they are the focus of scientific research, across a wide range of biological disciplines. Some organisms – mostly plants and vertebrates – are over-represented in various scientific fields^7, 9, 10^, are more likely to raise funds^11^, or are considered ecologically more important than others^12^. It has been shown, however, that focusing on a few, often charismatic, species, prevents reaching global conclusions and developing efficient conservation plans^7, 13, 14^. Rare, small or uncharismatic creatures do play pivotal functions in ecosystems^15, 16^. In addition, biomimicry, i.e. the application of the properties of living organisms to technology, and bioprospecting activities, i.e. the search for new natural products in wild species, cannot be performed efficiently when less than 1% of known species have been carefully studied^17^. Thus, given its scientific and societal significance, describing taxonomic bias in the study of biodiversity and understanding its underlying causes are undeniable priorities.

Taxonomic bias in science has long been recognized^10, 18, 19^ but its origin is less clear. Obviously, some organisms are more difficult to study than others because they live in remote habitats, are local endemics or are microscopic and difficult to identify^20^. But these intrinsic features alone cannot fully explain the pervasive taxonomic bias observed in science. Two hypotheses on the role of two extrinsic factors can then be put forward: the ‘taxonomic research’ hypothesis and the ‘societal preferences’ hypothesis. The ‘societal preferences’ hypothesis suggests that societal interests influence and bias the choice of study organisms^21, 22^. The ‘taxonomic research’ hypothesis implies that scientific reasons and limitations will lead and orientate biodiversity data gathering^20^. Because of the intricate interactions between scientists, citizens and funding agencies, and their mixed influence^23^, the underlying mechanisms are unclear. Nevertheless, these hypotheses deserve to be explored and confronted at a global taxonomic scale. Moreover, the recent development of citizen science^24^ may have increased the impact of societal preferences. Here, to investigate the relative impact of ‘societal preferences’ and ‘taxonomic research’ on biodiversity data, we used the number of webpages from Bing searches and the number of publications retrieved from Web of Science, as proxies, respectively (see Methods).

The study of biodiversity is a daunting task – ca. 10 million species are estimated to inhabit the planet – that requires deploying a considerable workforce to gather and analyse biodiversity data^25^. Fortunately, for ethical and scientific reasons^26–28^, data sharing practices and tools like the Global Biodiversity Information Facility (GBIF) were developed, facilitating access to species occurrence records. The GBIF portal was chosen because it hosts the biggest open access primary biodiversity database and, even though the big data paradigm does not mean that big datasets are devoid of flaws, they offer a significant potential for new and broad insights^29^. Moreover, although open access primary biodiversity data are heterogeneous, resulting from the good will of contributors and not from a well-planned sampling protocol^30^, they reflect our knowledge and practices in the study of biodiversity. Thus, they can be used to investigate taxonomic bias on a large geographical and taxonomic scale.

Here, we aim to quantify taxonomic bias in biodiversity data using 626 million of GBIF-mediated occurrences covering 24 classes of organisms. After careful data validation procedures, we characterized biodiversity gaps, a necessary first step before trying to bridge these gaps^31^. We did not assess the validity of GBIF mediated data, which is an issue that has already been raised and discussed repeatedly^32–36^. Instead, we quantified taxonomic bias and imprecision (i.e. when an occurrence has been identified not at the species level but only at a higher taxonomic level) and related them to information provided in the occurrence records information (data origin, record date and locality). We tested the relative impact of societal preferences and taxonomic research on taxonomic bias, using public interest (i.e. the number of webpages) and taxonomic research quantity (i.e. the number of publications) as explaining variables, respectively. Opposing these hypotheses enabled us to suggest future directions for developing strategies for representative sampling of biodiversity while this is still possible.

## Results

### Global taxonomic coverage and taxonomic precision

24 classes of organisms recorded in the GBIF database had more than 1 million occurrences, with widely variable numbers of occurrence recordings (Table 1). More than half of the records were bird (Aves) occurrences (345 million occurrences; 53% of the GBIF mediated data), even though birds represent only 1% of the total number of species catalogued in GBIF. Aves was also the class with the highest median number of occurrences per species (med_/sp_ = 371). By contrast, and despite being three times more speciose, Arachnida had only 2.17 million occurrences and one of the lowest median numbers of occurrences per species (med_/sp_ = 3). The lowest values of the median number of occurrences per species (i.e. below 7) were found for several classes of Arthropods (Insecta, Maxillopoda, Arachnida, Malacostraca), some fungi (Agaricomycetes) and diatoms (Bacillariophyceae). Magnoliopsida and Insecta, two highly speciose classes, were the ones with the highest number of species recorded. Only six of the 24 classes had a median number of occurrences per species higher than 20.

**Table 1:** Biodiversity occurrence data statistics for 24 taxonomic classes.

	nb_occ_ (millions)	p_>1_ (thousands)	med_/sp_ (mad)	Taxonomic precision
**Aves**	345.11	12.82	371 (541)	0.99
**Magnoliopsida**	118.21	261.01	19 (25)	0.92
**Insecta**	46.78	352.78	3 (3)	0.77
Liliopsida	36.75	68.99	15 (19)	0.95
Actinopterygii	14.18	30.73	27 (37)	0.92
**Mammalia**	10.78	11.53	15 (21)	0.88
Bryopsida	6.06	18.85	7 (9)	0.95
Gastropoda	5.85	46.99	7 (9)	0.69
**Reptilia**	4.98	11.30	24 (34)	0.88
**Lecanoromycetes**	4.97	17.79	8 (10)	0.93
Polypodiopsida	4.91	12.65	23 (31)	0.95
**Amphibia**	3.94	5.89	54 (76)	0.91
**Agaricomycetes**	3.80	23.53	4 (4)	0.93
Malacostraca	2.73	30.16	6 (7)	0.73
Globothalamea	2.68	4.07	10 (13)	0.74
Arachnida	2.17	38.11	3 (3)	0.77
Bivalvia	2.02	14.02	9 (12)	0.70
Bacillariophyceae	1.96	11.19	2 (1)	0.70
Maxillopoda	1.87	9.98	4 (4)	0.58
Pinopsida	1.57	0.91	110 (160)	0.95
Jungermanniopsida	1.41	6.93	7 (9)	0.91
Polychaeta	1.29	8.77	6 (7)	0.73
Florideophyceae	1.07	5.78	17 (24)	0.88
Anthozoa	1.03	8.64	7 (9)	0.59
*Total for 24 classes*	*626.13*	*1013.39*	*7 (9)*	*0.94*
*Total in the GBIF*	*649.79*	*1200.38*	*6 (7)*	*0.93*

The number of occurrences (nb_occ_) was obtained before the use of any filter. The number of species (p_>1_) corresponds to the number of unique scientific names having at least one occurrence. In bold are the eight classes selected to study the taxonomic bias at the ordinal level. med_/sp_ is the median number of occurrences per species and mad is the associated median deviation. Taxonomic precision is the proportion of taxa identified at least at the species level.

With regard to taxonomic precision, 94% of GBIF occurrences were identified (at least) at the species level (88% not counting Aves). The lowest levels of taxonomic precision were found in Maxillopoda and Anthozoa (58% and 59% of occurrences, respectively), whereas the highest levels were found in the different classes of Plantae (91 to 95% of occurrences in Magnoliopsida, Liliopsida and Pinopsida), Fungi (93% in Agaricomycetes and Lecanoromycetes) and Aves (99%).

### Taxonomic bias

**Figure 1:**
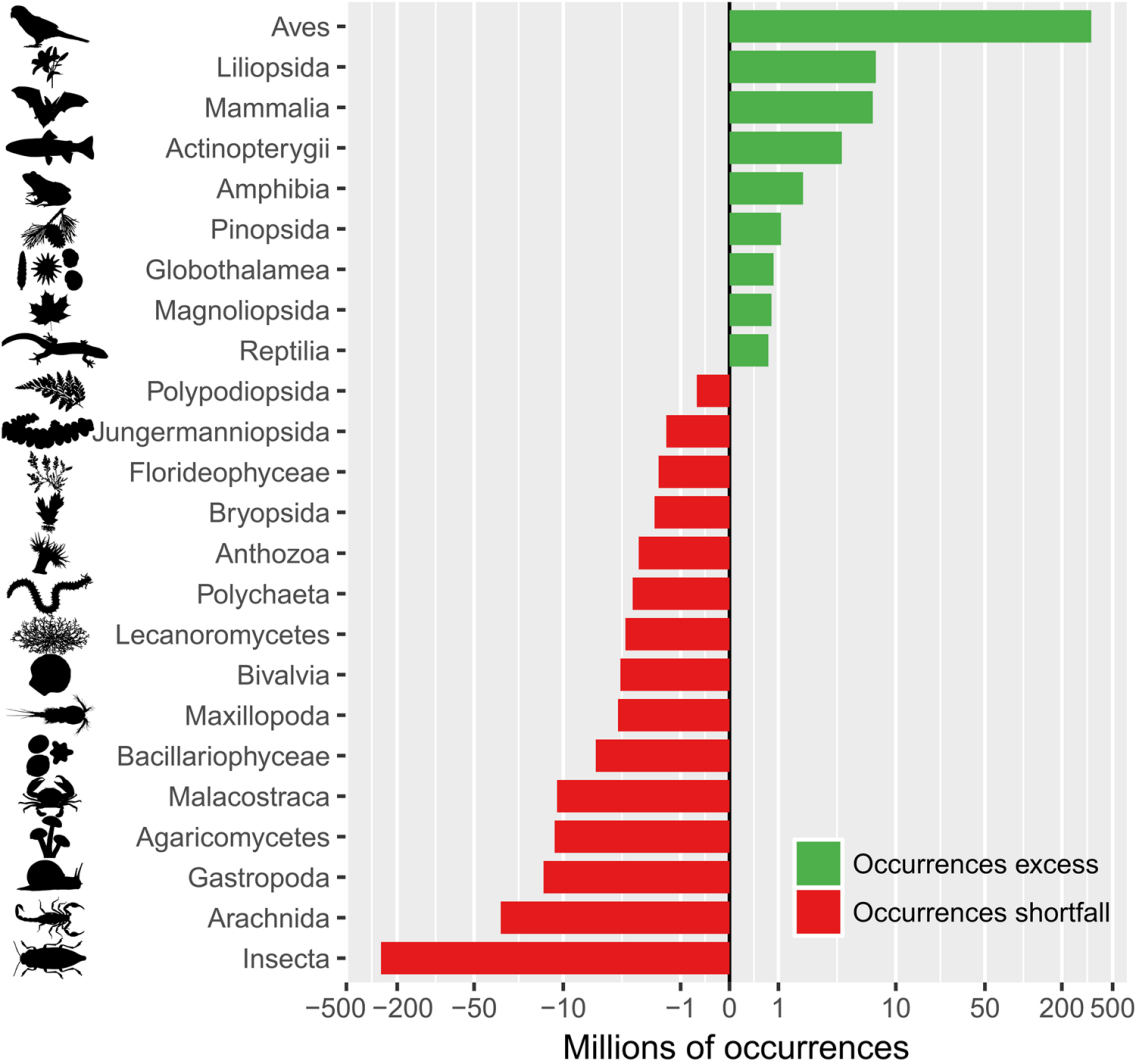
Taxonomic bias in biodiversity occurrence data. The vertical line at x = 0 depicts the ‘ideal’ number of occurrences per class, where each class is sampled proportionally to its number of known species. Green and red bars show the classes that are over- and under-represented in the GBIF mediated database compared to this ‘ideal’ sampling, respectively. Insects lack >200 millions occurrences and birds have an excess of >200 millions occurrences compared to an unbiased taxonomic sampling. Because birds and insects are greatly over- and under-represented, respectively, an inverse hyperbolic sine transformation was used for the x-axis.

**Figure 2:**
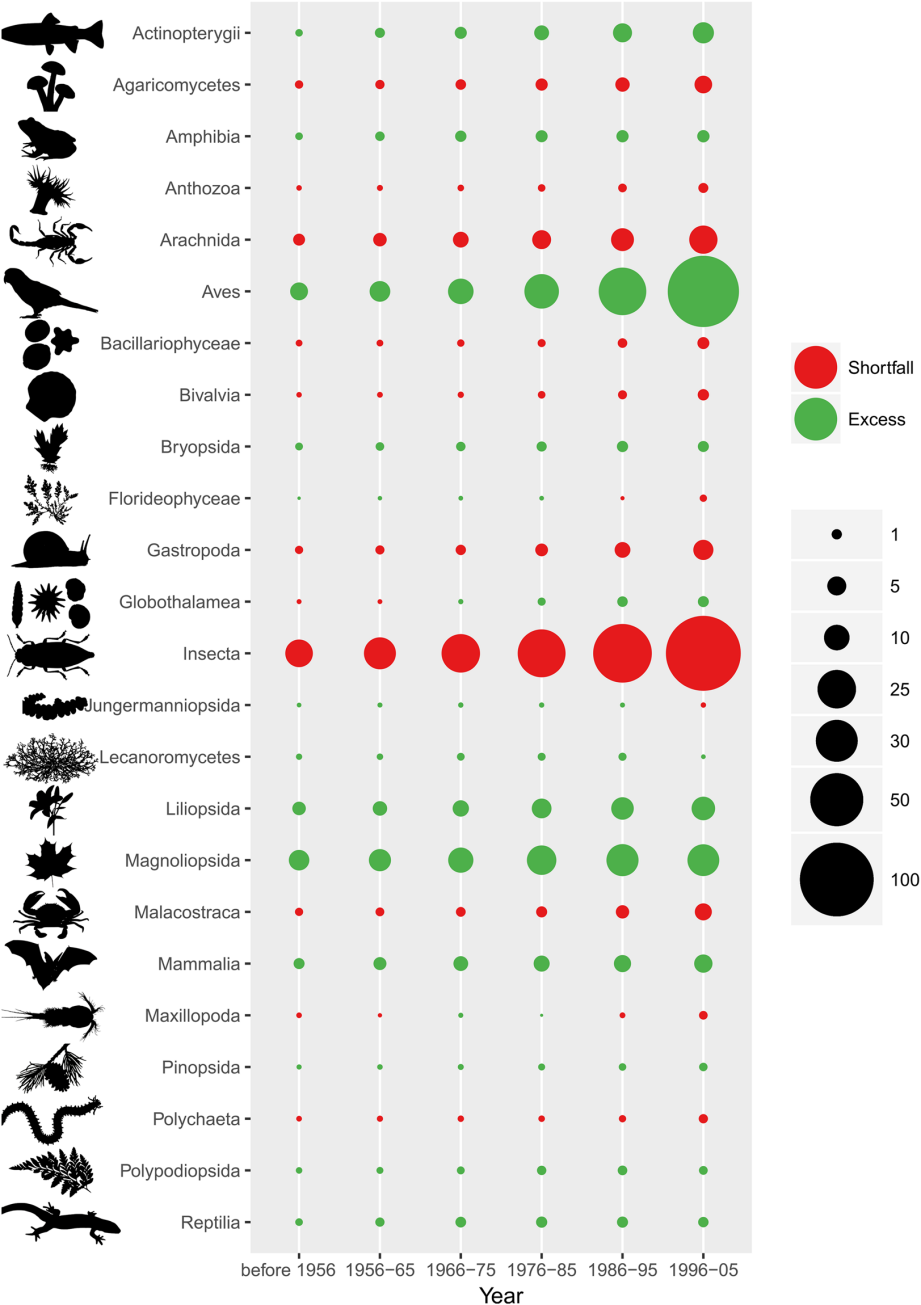
Evolution over time of the taxonomic bias for each class. The larger the circle, the higher the deviation from I, the ‘ideal’ number of occurrences per class if no taxonomic bias is observed. Red dots indicate negative deviations (i.e. shortfall in occurrences = under-represented classes); green dots indicate positive deviations (i.e. excess of occurrences = over-represented classes).

**Figure 3:**
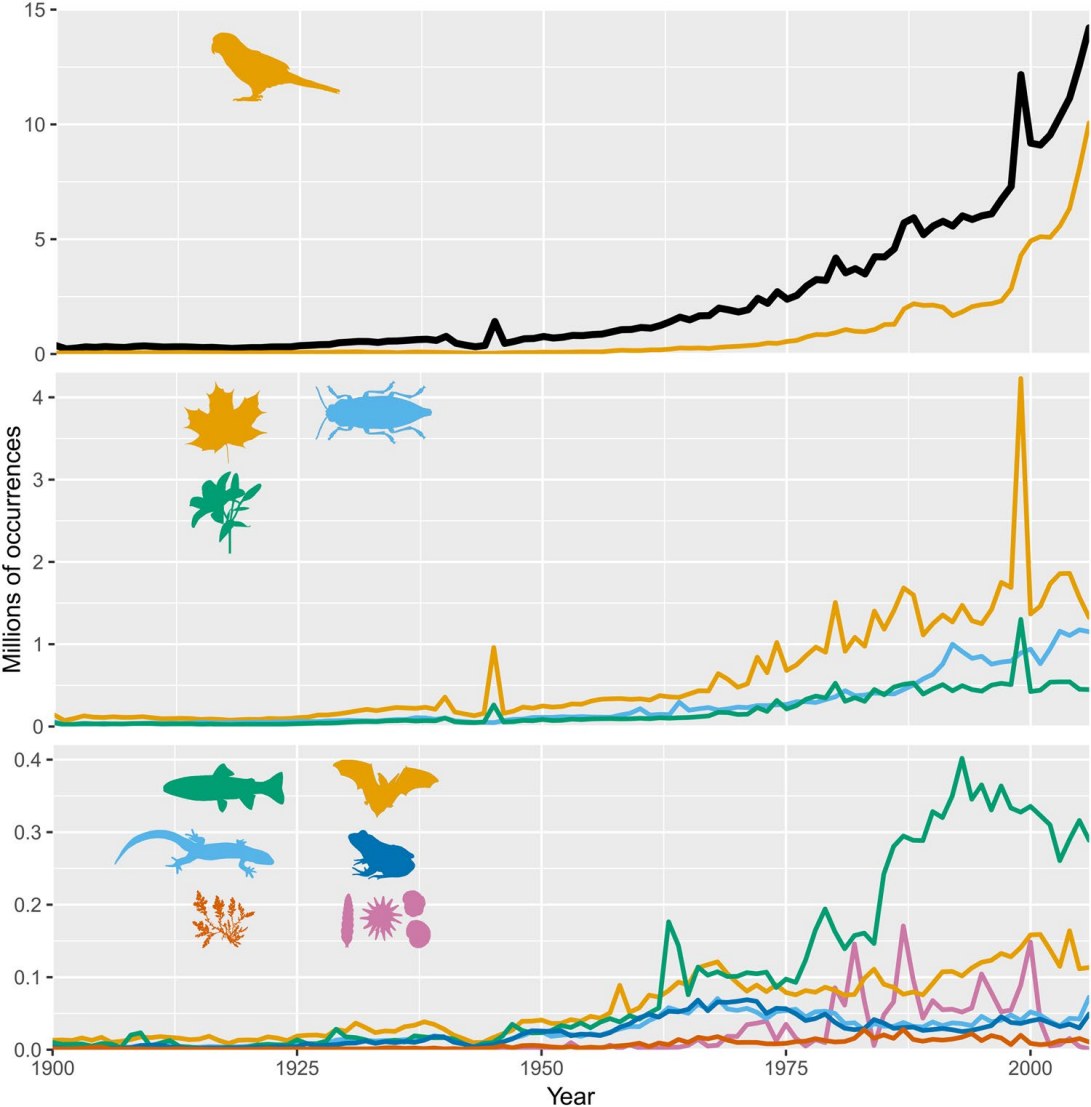
Biodiversity occurrences recorded in GBIF between 1900 and 2006. For each curve, the number of occurrences was plotted yearly. *Top*: black = all 24 classes considered together, yellow = Aves; *Middle*: yellow = Magnoliopsida, blue = Insecta, green = Liliopsida; *Bottom*: green = Actinopterygii, yellow = Mammalia, light blue = Reptilia, dark blue = Amphibia, orange = Florideophyceae, purple = Globothalamea.

Of the 2.2 million of species referenced in the GBIF taxonomic backbone, 1.2 million species can be found in the GBIF published datasets and 1.01 million belong to the 24 classes selected here. The number of recorded species per class was not proportional to their known species richness, highlighting a strong taxonomic bias. Aves and Insecta were, by far, the most over- and under-represented classes, respectively. Mammalia, Liliopsida, Actinopterygii, Amphibia and Magnoliopsida were also over-represented, whereas Arachnida, Gastropoda, Agaricomycetes, Malacostraca and Bacillariophyceae were under-represented (Fig. 1 and Supplementary Fig. 1). This taxonomic bias was already apparent more than 50 years ago, meaning that classes that were over- or under-represented in the 1950′s are still over- or under-represented today (Fig. 2). Nonetheless, we found an increase in taxonomic bias over time, mostly due to the faster accumulation of data for birds compared to other classes (Fig. 3 *top*; 283 million bird occurrences recorded between 2000 and 2016). Recently, data has accumulated faster than ever before for most classes (Fig. 3 *top, middle* and Supplementary Fig. 2) however, for Amphibia, Reptilia and Florideophyceae, the number of occurrences recorded per year has stagnated or even declined over the past 40 years (Fig. 3 *bottom*).

**Figure 4:**
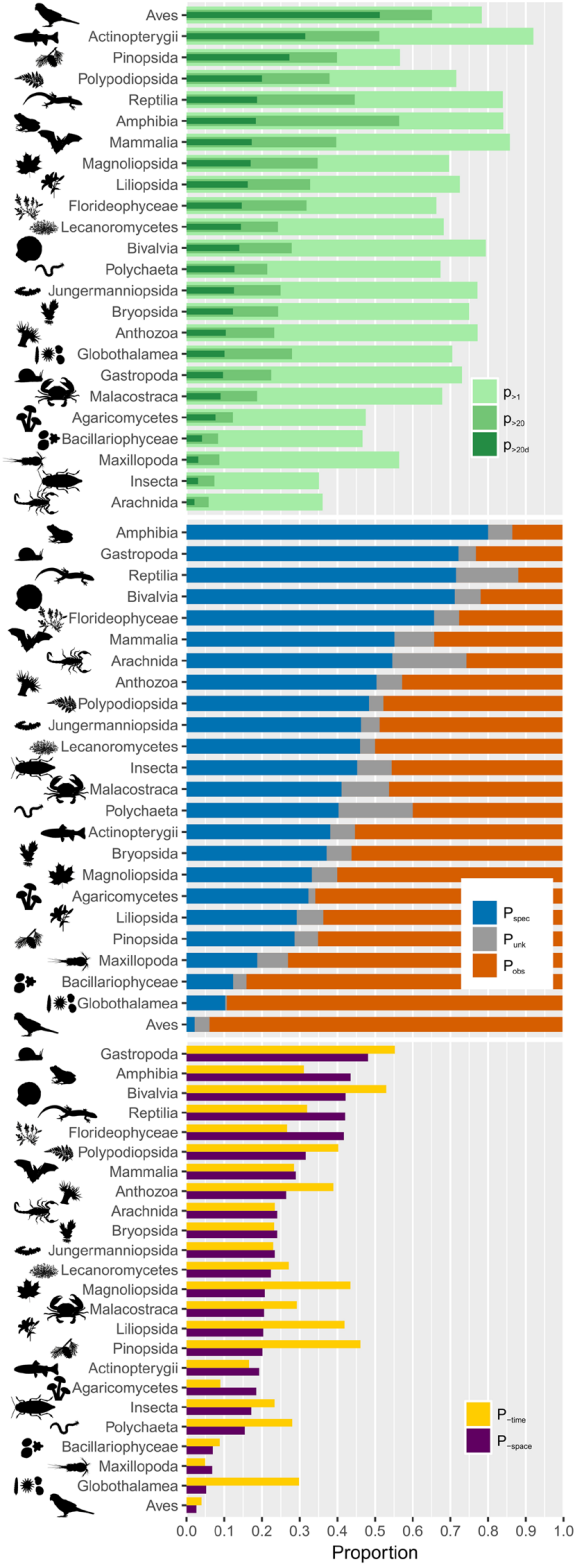
Taxonomic heterogeneity in sampling, occurrence data origin and quality for 24 taxonomic classes. *Top*: Proportion of species per class recorded in GBIF with at least one occurrence (light green: p_>1_), with more than 20 occurrences (green: p_>20_), and with more than 20 spatially distinct occurrences (i.e. “decently” sampled – dark green: p_>20d_). For all classes, except Aves, less than 1/3 of all species are “decently” sampled. Classes are ranked according to their proportion of “decently” sampled species. *Middle* **:** Occurrence origin (*basisOfRecord*) for each class. Some classes like Amphibia have a high proportion of occurrences based on specimens (blue: living or preserved specimen, material samples or fossils), whereas others like Aves have a majority of occurrences based on observation (orange: machine or human observation, literature). Grey bars show occurrences where the record basis is unknown. Classes are ranked according to their proportion of specimen-based occurrences. *Bottom* **:** Data incompleteness. Proportion of occurrences with spatial (purple) or temporal (yellow) inaccuracies for each class. Spatial inaccuracy corresponds to an occurrence lacking coordinates or tagged has having geospatial issues by GBIF. Temporal inaccuracy corresponds to a sampling event with no specified month or year. Classes are ranked according to their proportion of occurrences with spatial issues.

Twenty out of 24 classes had more than 50% of their described species referenced at least once in GBIF, and, for 14 of these classes, these statistics rose to 70% or more. By contrast, only 35% of Insecta and 36% of Arachnida species were referenced at least once in GBIF (Fig. 4 *top*). Furthermore, species were more or less intensely recorded in GBIF: 21% had only one occurrence (i.e. 212,911 species), 44% had between 2 and 19 occurrences (i.e. 446,643 species), and 35% had 20 or more occurrences (i.e. 353,843 species). This density of recordings per species was unevenly distributed between classes (Fig. 4 *top*). Only three classes (Aves, Amphibia and Actinopterygii) had more than half of their species with at least 20 occurrences, and only Aves had more than half of its species “decently” sampled (i.e. with 20 spatially distinct occurrences). This contrasted strikingly with the Arthropod classes, where, at best, 9% of species were “decently” sampled, even though Malacostraca had 68% of its species recorded in the GBIF.

This taxonomic bias recurs at a lower taxonomic scales. We selected eight classes and showed that, for all of them, some orders were better represented in the GBIF-mediated database than others (Table 2 and Supplementary Table S1). For instance, the median number of occurrences varied largely within each class, some orders having medians that were more than 50 times higher than those of other orders of the same class (e.g. m_Phaethontiformes_ = 5504 *vs* m_Sphenisciformes_ = 2; m_Chiroptera_ = 107 *vs* m_Cetacea_ = 2). The smallest difference in medians was found within poorly represented classes, in which all orders have medians less than 20. Taxonomic precision was also estimated and found to be highly heterogeneous between orders of the same class. The largest differences were observed within Insecta. More than 90% of occurrences were identified at the species level for four orders (Siphonaptera, Odonata, Orthoptera and Psocodea), whereas taxonomic precision ranged from 35 to 0.5% for Grylloblattodea, Mantophasmatodea and Strepsiptera. Taxonomic precision within Mammalia was also very heterogeneous ranging from 22% (Perissodactyla) to 99% (Monotrema and Notoryctemorphia). Conversely, taxonomic precision was less variable between orders of Lecanoromycetes (over 89% taxonomic precision for all orders), Magnolopsida (82% and above) and Aves (77% and above).

**Table 2:** Biodiversity occurrence data statistics for the orders (maximum 10) with the most occurrences within eight selected classes.

	Order	nb_occ_ (millions)	p > 1 (thousands)	med/sp (mad)	Taxonomic precision		Order	nb_occ_ (millions)	p > 1 (thousands)	med/sp (mad)	Taxonomic precision
Agaricomycetes	Agaricales	1.80	14.14	4 (4)	0.93	Lecanoromycetes	Lecanorales	2.86	9.24	8 (10)	0.93
	Russulales	0.53	1.96	7 (9)	0.94		Teloschistales	0.75	2.38	11 (15)	0.94
	Polyporales	0.53	2.72	4 (4)	0.92		Peltigerales	0.58	1.45	15 (21)	0.92
	Hymenochaetales	0.27	0.74	7 (9)	0.91		Pertusariales	0.26	1.10	7 (9)	0.91
	Boletales	0.25	1.39	4 (4)	0.90		Ostropales	0.20	2.75	5 (6)	0.90
	Cantharellales	0.10	0.59	4 (4)	0.98		Umbilicariales	0.09	0.14	21 (30)	0.98
	Thelephorales	0.08	0.41	5 (6)	0.97		Baeomycetales	0.08	0.16	15 (21)	0.97
	Auriculariales	0.05	0.26	4 (4)	0.98		Candelariales	0.07	0.08	19 (27)	0.98
	Gomphales	0.04	0.30	7 (9)	0.92		Acarosporales	0.05	0.35	6 (7)	0.92
	Corticiales	0.02	0.25	4 (4)	0.95		Agyriales	0.01	0.06	12 (16)	0.95
Amphibia	Anura	2.85	5.03	54 (76)	0.82	Magnoliopsida	Asterales	17.02	33.12	16 (21)	0.82
	Caudata	1.06	0.60	172 (246)	0.93		Lamiales	13.03	28.01	17 (22)	0.93
	Gymnophiona	0.02	0.16	14 (19)	0.89		Fabales	11.27	24.13	25 (34)	0.89
							Caryophyllales	10.14	15.14	17 (22)	0.96
Aves	Passeriformes	185.57	7.34	368 (525)	0.95		Rosales	9.45	13.76	12 (16)	0.95
	Charadriiformes	37.63	0.48	2538 (3760)	0.94		Malpighiales	7.06	19.68	22 (30)	0.94
	Anseriformes	34.12	0.21	5135 (7609)	0.93		Gentianales	5.06	21.70	17 (22)	0.93
	Accipitriformes	14.83	0.34	579 (855)	0.95		Ericales	4.74	14.07	20 (27)	0.95
	Piciformes	13.81	0.48	467.5 (650)	0.93		Myrtales	4.63	14.71	26 (34)	0.93
	Columbiformes	11.41	0.38	261 (366)	0.93		Apiales	4.55	5.93	20 (27)	0.93
	Pelecaniformes	11.01	0.16	1517 (2248)	0.90	Mammalia	Rodentia	3.62	3.59	25 (36)	0.90
	Gruiformes	4.98	0.28	148.5 (219)	0.94		Chiroptera	2.23	1.31	107 (154)	0.94
	Suliformes	4.57	0.09	777 (1150)	0.93		Carnivora	1.62	0.89	10 (13)	0.93
	Apodiformes	4.17	0.53	565 (802)	0.97		Diprotodontia	0.63	0.22	48.5 (70)	0.97
Insecta	Lepidoptera	17.41	64.11	3 (3)	0.76		Artiodactyla	0.63	0.98	8 (10)	0.76
	Coleoptera	9.77	96.27	3 (3)	0.93		Soricomorpha	0.48	0.67	16 (22)	0.93
	Hymenoptera	8.23	58.02	3 (3)	0.88		Cetacea	0.40	0.54	2 (1)	0.88
	Diptera	4.70	63.99	2 (1)	0.90		Lagomorpha	0.28	0.19	20.5 (29)	0.90
	Hemiptera	1.97	33.81	2 (1)	0.22		Perissodactyla	0.20	0.52	9 (12)	0.22
	Trichoptera	1.27	6.69	3 (3)	0.78		Primates	0.12	0.80	12 (16)	0.78
	Odonata	1.20	3.48	11 (15)	0.90	Reptilia	Squamata	4.49	9.16	37 (52)	0.90
	Orthoptera	0.96	9.57	3 (3)	0.78		Testudines	0.37	0.63	16 (22)	0.78
	Ephemeroptera	0.40	1.27	4 (4)	0.61		Crocodylia	0.05	0.16	3 (3)	0.61
	Plecoptera	0.23	1.92	4 (4)	0.88		Rhynchocephalia	0.00	0.02	2 (1)	0.88

Statistics and abbreviations as in Table 1.

### Explanatory variables

In GBIF, recorded occurrences can refer to a collected specimen (or object) or an observation. The proportion of specimen- *vs* observation-based occurrences differed greatly between classes (Fig. 4 *middle*). Some classes had 90% or more of their occurrences based on observation (e.g. Globothalamea, Aves), whereas others had between 70 and 80% of occurrences based on specimens (e.g. Amphibia, Gastropoda, Reptilia and Bivalvia). Between these extremes, the relative proportion of specimen- vs observation-based occurrences in the 24 classes formed a continuum, with a few classes having an almost equivalent number of occurrences of both origins (e.g. Insecta). Three of the four groups of Tetrapods (Amphibia, Reptilia and Mammalia) had occurrences based mainly on specimens, whereas birds had the highest proportion of observation-based occurrences (94%).

Although temporal and geographical information can also be added to a record, these fields are informed with more or less precision. The proportion of spatial and temporal inaccuracies (p_-time_ and p_-space_) differed greatly between classes (Fig. 4 *bottom*). Only 4% of Aves occurrences had temporal and/or spatial inaccuracies, whereas 48% and 55% of Gastropoda occurrences had spatial and temporal inaccuracies, respectively. Along with Gastropoda, the classes with the highest inaccuracy rates were Amphibia, Bivalvia and Reptilia, and these four classes were the ones with the highest proportion of specimen-based occurrences.

**Figure 5:**
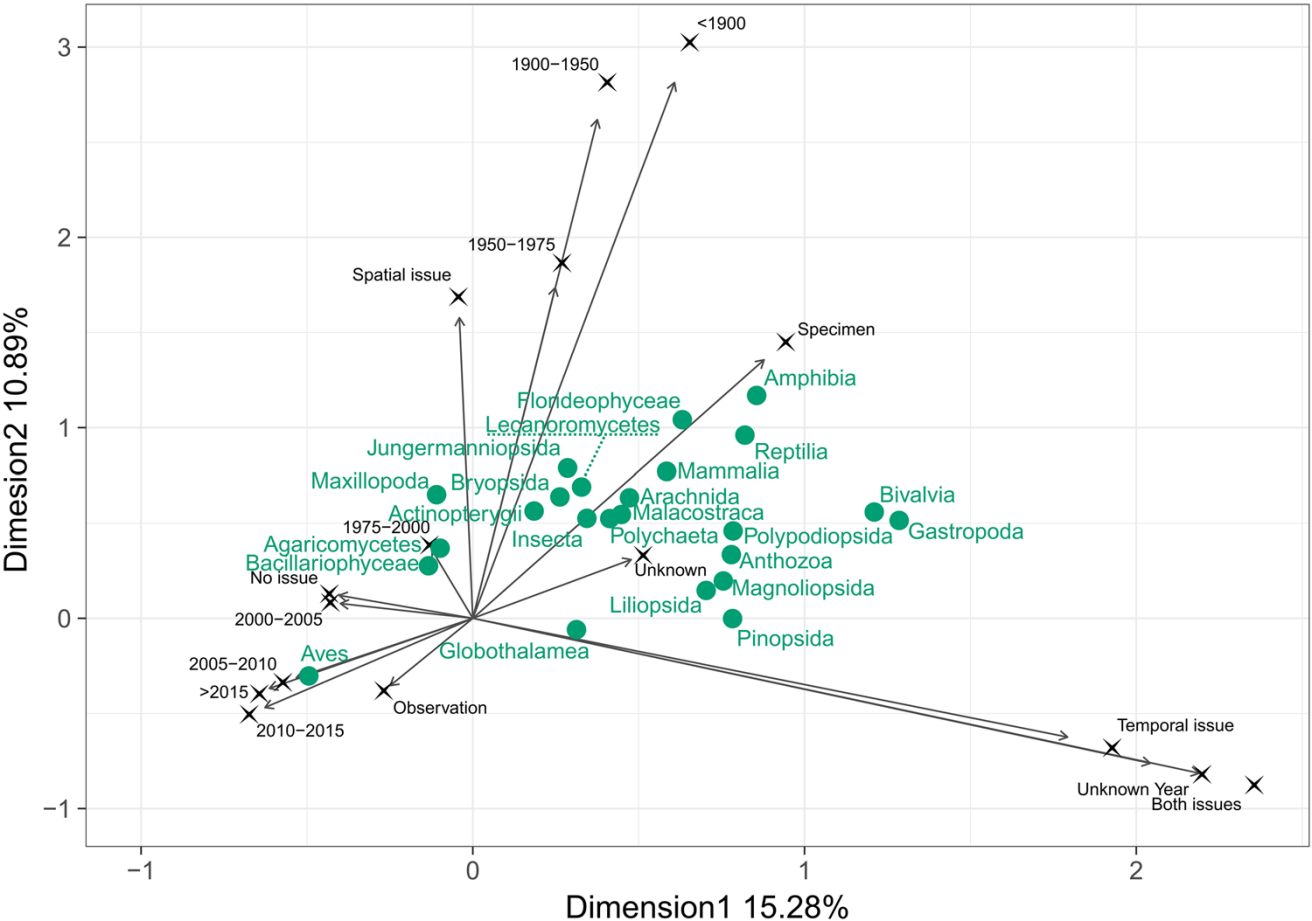
Relation between age, origin and quality of the occurrence data for 24 taxonomic classes. Graph showing the first two axes of a Multiple Correspondence Analysis (MCA) performed on 5 million random occurrences. Labels in black represent the categories considered for all occurrences. Classes’ names (in green) are placed at the average position of the class occurrences. Occurrence age contains eight time intervals and an Unknown Year category; data origin contains three categories: *Specimen* for specimen-based occurrences, *Observation* for observation-based occurrences, and *Unknown* for unknown origins; data quality contains four categories: *Temporal issue* for the lack of year or month, *Spatial issues* for the lack of coordinates, *Both issues* and *No issue*.

All Multiple Correspondence Analyses (MCA) showed that occurrences recorded before 1975 were grouped with specimen-based occurrences and with occurrences with spatial issues (Fig. 5). Conversely, more recent occurrences were grouped with complete and observation-based occurrences. Most of the classes, and in particular Amphibia, Reptilia and Florideophyceae, were in the upper right section of the graph (old, incomplete specimen-based occurrences), whereas Aves was in the lower left section, characterized by recent and complete observations.

Public interest (inferred from the number of web pages referenced by a search engine) and taxonomic research effort (inferred from the number of publications in Web of Science) were assessed and used in Generalized Linear Models (GLM). The number of web pages (with the keyword “species” added to the species’ scientific name) ranged from 0 to 1.8 million with a median number of 1,480 pages for the 24,000 best-represented species (1,000 species for each class) and 22 pages for the randomly chosen species. The number of publications, tallied for 453 orders, ranged from 0 (for eight orders) to 72 426 for Coleoptera, with a median number of 229 publications.

For most classes, GLMs suggested a positive and significant correlation between public interest and the number of occurrences in GBIF (Table 3). A few negative correlations were found but were never significant. The quantity of research was not significantly correlated with the number of occurrences for most classes, and, when the correlation was significant, it was either positive (e.g. Mammalia) or negative (e.g. Agaricomycetes). A significant correlation between public interest and research quantity was found in 10 out of 47 cases.

**Table 3:** GLM results assessing the link between research quantity, public interest and their combined interaction on the amount of biodiversity data per class.

Class	Selected species	Nb species	Public interest influence pval	Research influence pval	Interaction influence pval
Actinopterygii	Best	930	(+) 0.000*	(+) 0.780	(-) 0.023*
	Random	883	(+) 0.000*	(+) 0.004*	(-) 0.014*
Agaricomycetes	Best	951	(+) 0.000*	(-) 0.002*	(+) 0.055
	Random	738	(+) 0.000*	(-) 0.032*	(+) 0.659
Amphibia	Best	916	(+) 0.573	(-) 0.000*	(+) 0.058
	Random	875	(+) 0.076	(+) 0.024*	(-) 0.714
Anthozoa	Best	910	(+) 0.304	(-) 0.273	(+) 0.000*
	Random	744	(+) 0.002*	(-) 0.101	(+) 0.198
Arachnida	Best	930	(-) 0.376	(-) 0.021*	(+) 0.000*
	Random	799	(+) 0.029*	(-) 0.624	(-) 0.632
Aves	Best	930	(-) 0.376	(-) 0.021*	(+) 0.000*
	Random	850	(+) 0.000*	(+) 0.182	(+) 0.277
Bacillariophyceae	Best	885	(+) 0.000*	(-) 0.616	(+) 0.174
	Random	780	(+) 0.000*	(-) 0.011*	(-) 0.230
Bivalvia	Best	928	(+) 0.000*	(-) 0.082	(+) 0.160
	Random	755	(+) 0.000*	(+) 0.313	(-) 0.087
Bryopsida	Best	905	(+) 0.000*	(+) 0.000*	(-) 0.079
	Random	846	(+) 0.000*	(+) 0.366	(-) 0.672
Florideophyceae	Best	904	(+) 0.000*	(-) 0.070	(+) 0.000*
	Random	818	(+) 0.000*	(+) 0.002*	(+) 0.665
Gastropoda	Best	718	(+) 0.683	(+) 0.183	(+) 0.045*
	Random	521	(+) 0.033*	(+) 0.110	(-) 0.738
Globothalamea	Best	886	(+) 0.005*	(+) 0.000*	(-) 0.599
	Random	793	(+) 0.015*	(-) 0.310	(+) 0.106
Insecta	Best	967	(+) 0.000*	(-) 0.246	(-) 0.216
	Random	769	(+) 0.013*	(+) 0.369	(-) 0.601
Jungermanniopsida	Best	905	(+) 0.000*	(+) 0.405	(+) 0.013*
	Random	850	(+) 0.001*	(+) 0.999	(+) 0.558
Lecanoromycetes	Best	961	(+) 0.000*	(-) 0.667	(-) 0.851
	Random	804	(+) 0.000*	(-) 0.584	(+) 0.560
Liliopsida	Best	931	(+) 0.000*	(+) 0.060	(-) 0.168
	Random	856	(+) 0.000*	(+) 0.000*	(+) 0.615
Magnoliopsida	Best	959	(+) 0.000*	(+) 0.003*	(-) 0.205
	Random	768	(+) 0.001*	(-) 0.170	(+) 0.863
Malacostraca	Best	906	(+) 0.000*	(-) 0.002*	(-) 0.001*
	Random	757	(+) 0.156	(-) 0.392	(+) 0.154
Mammalia	Best	913	(+) 0.000*	(+) 0.024*	(-) 0.000*
	Random	800	(+) 0.000*	(+) 0.049*	(-) 0.100
Maxillopoda	Best	889	(+) 0.000*	(+) 0.017*	(-) 0.540
	Random	835	(+) 0.012*	(-) 0.898	(+) 0.510
Pinopsida		796	(+) 0.000*	NA	NA
Polychaeta	Best	790	(+) 0.000*	(-) 0.053	(+) 0.389
	Random	712	(+) 0.010*	(-) 0.212	(+) 0.519
Polypodiopsida	Best	938	(+) 0.000*	(-) 0.174	(+) 0.335
	Random	785	(+) 0.000*	(+) 0.048*	(-) 0.473
Reptilia	Best	940	(+) 0.180	(+) 0.627	(+) 0.190
	Random	794	(+) 0.040*	(+) 0.104	(+) 0.448

A positive correlation between public interest and the number of occurrences was found in most classes. Values followed by * have a significant p-value at a 5% threshold. (+) indicates a positive influence while (-) indicates a negative influence of the variable on the number of occurrences. Nb species = number of species used in the GLM after removing outliers; pval = p-values; NA = not available (because no order information and therefore no research quantity was available for Pinopsida).

## Discussion

Taxonomic bias, i.e. the fact that some taxa are more investigated than others, is a well-known problem for the study of biodiversity. How can we infer general principles and put in place effective strategies for biodiversity conservation when some taxa are over-studied while others are ignored? Although known for a long time, taxonomic bias is currently receiving an increasing attention. However most studies on taxonomic bias have been restricted to a few taxa or areas^9, 19, 30, 37–39^. By analysing data from the biggest biodiversity data repository available, we emphasize here the prevalence of taxonomic bias in biodiversity data.

Unsurprisingly, and as previously reported regarding GBIF mediated data^33^, we show that birds are over-represented in biodiversity data. Some studies highlighted the over-representation of birds in diverse disciplines ranging from behavioural ecology to evolution and conservation^9, 40^. The ever-growing number of observations that bird enthusiasts report undoubtedly amplify bias. Other vertebrate classes (Actinopterygii and Mammalia, and to a lesser extent Reptilia and Amphibia) are relatively well represented in the GBIF-mediated database, as are most Plantae classes, especially Liliopsida and Magnoliopsida. On the other hand, Arthropods (Insecta, Arachnida, Malacostraca and Maxillopoda) and Mollusca (Gastropoda and Bivalvia) are under-represented, with insects being particularly mis-represented. Birds and insects are obvious outliers but, beyond these two classes, the taxonomic bias in biodiversity data remains blatant.

Taxonomic bias is even more apparent when considering “decently” sampled species, namely species sampled in at least 20 different points on the globe. For any study requiring a number of different sampling points, like those relying on niche modelling, the field of investigation is restricted to vertebrates and plants on land and Actinopterygii in aquatic habitats. Invertebrates and fungi, on the other hand, have to be virtually ignored because of insufficient data at the scale of the planet. Given that these neglected organisms have a high diversity and play crucial roles in diverse ecosystems^2, 3, 15^, this situation will inevitably result in an unbalanced fundamental knowledge of biodiversity, risky guesses and uninformed conservation decisions^7, 14, 19, 41, 42^. A similar taxonomic bias, with equivalent outcomes, is found between orders within each class.

More disturbingly, we show that the taxonomic bias in biodiversity data, although known for a few decades^18^, has remained broadly the same since the 1950’s. The evolution of taxonomic bias over time has rarely been investigated, and never at a large taxonomic scale. Bonnet *et al*.^9^, focusing on vertebrates, showed there had been no changes in taxonomic chauvinism in ecology and behavioural research. Similarly, Stahlschmidt^21^ reported a static taxonomic bias from 2001 to 2010 in parental care research. He noted, however, that the absolute number of publications on parental care in birds increased significantly over this period. Along the same lines, Di Marco *et al*.^8^ emphasized that, in conservation science, some historically under-studied taxa were receiving more attention today, but underlined that a taxonomic bias toward taxa that are threatened or less rich in biodiversity still exists. Our results confirm this *status quo* situation at a larger taxonomic scale: most classes that were under- or over-represented in the GBIF mediated database in 1950 are still under- or over-represented today. Even though most classes are better recorded today than before, the gap between birds and the rest of biodiversity (i.e. ∼99% of known biodiversity) increases with time because bird occurrences accumulate much faster than other class occurrences. Thus, while most of biodiversity remains to be described^25^, the same taxa are preferentially studied and recorded over and over again.

The large taxonomic scale approach we used here comes with a few limitations. First, it must be emphasized that big datasets, like all sampling, are biased so that conclusions must be drawn accordingly^29^. Second, this large-scale approach implies that each species is equivalent and directly comparable, which is obviously arguable. Third, it neglects scale effects: species richness in insects is so large that whatever the means used, this class is always at risk of being understudied. Still, this approach enabled us to highlight the pervasiveness of taxonomic bias and bring new insights into the nature of this bias.

The underlying causes of taxonomic bias must be identified if one wants to reverse it. We suggest here that societal preferences, and not taxonomic research, orientate which biodiversity data are gathered. The most popular species on the web are also the species with the most records in GBIF. Moreover, the best-supported model, where the interaction between taxonomic research effort and the number of web pages was taken into account, indicated a significant effect of public interest on biodiversity data gathering. The role played by the general public in the study and conservation of biodiversity has already been established: positive links exist between public opinion, scientific productions and conservation policies, however the directionality of these interactions remains unclear^23, 43^. Our analyses confirm these interactions but do not allow us to clarify the causality issue. Although inevitable biases occur when using internet searches, such as the inability to distinguish scientific web pages from other web pages, particularly at such a broad taxonomic scale, “*many (30–80%) web pages containing the scientific names of species have little or nothing to do with scientific research*“^22^ indicating that our results are presumably related to societal preferences. Surveys to determine public preferences could help counteract this issue but should be carried out at large taxonomic scales.

Studying invasive alien species, Wilson *et al*.^22^ concluded that “*the choice of research subject in biology reflects the interests of society*”. Because of public interest, and not specifically for their scientific interest, studies of ‘public-aware’ taxa are more likely to be funded and receive more funding^11, 23, 44^. Our results provide further evidence of this trend, highlighting the active role of the general public in biodiversity data collecting, given that, for instance, the biggest dataset was provided by eBird (211 million occurrences), a collective enterprise devoted to birds and partly relying on citizen science^45^. For multiple reasons (e.g. the difficulty of obtaining permits, more and more endangered species, citizen science programmes, population decline, etc.), less specimen-based occurrences are now reported. Amphibia, Gastropoda and Reptilia, the three classes with the highest proportion of specimen-based occurrences, are also the classes with a decreasing or stabilizing trend in data accumulation. We thus anticipate an increasing bias between taxa mostly known from observation-based occurrences and taxa mostly known from specimen-based occurrences. In addition, a lot of records are old and incomplete, and could soon, or already, be obsolete^46^, which risks reinforcing the taxonomic bias against classes with relatively few recent occurrences.

The good news is that the observed taxonomic bias can be corrected. Shine & Bonnet^47^ showed how snakes, which were under-represented in ecology among terrestrial vertebrates until 1990, have grown in popularity in this scientific field, illustrating that acting on taxonomic bias is possible. Similarly, for most classes, occurrences accumulate at a much faster rate now that 50 or 30 years ago, which is an encouraging trend. Obviously, this trend can also result from changes in data-sharing practices, and not simply from overall data collection. Still, as we are accumulating more and more biodiversity data, the question of how to efficiently sample the whole of biodiversity remains open. The biodiversity knowledge chain is complex and its links influence one another. Scientists play a key role in this chain. However, our results show that they alone cannot ensure that biodiversity is sampled adequately and that societal preferences are too important to be ignored. Scientists must reach out to the lay audience^22, 23, 48^ and advertise under-represented organisms to the general public. For instance, the crucial role of protists in ecosystem functioning probably seems too obscure to generate any interest from the general public^49^. New practices or methods, from citizen science to metagenomics, should also help increase public awareness and would have even more impact if programmes were developed jointly between science and society^20, 50^. The expected gain would be colossal and would achieve more than a well-balanced sampling of biodiversity: new vocations in science, more efficient citizen sciences programmes, influence on funding and political decisions, etc.

Citizen science and data gathering by non- professionals might be decisive in the near future. The contribution of citizen science to the most over-represented class of GBIF-mediated data, birds, dates back more than a hundred years^51^. Different fields of research from molecular engineering^52^ to quantum science^53^ and neurosciences^54^ have greatly benefited from the involvement of non-professionals, and it has been shown that a well-made citizen science programme can could produce in two years the same amount of data that scientists can produce in a decade^55^. Yet, the use of citizen science for studying taxa that are not as charismatic as birds or mammals is still in its infancy^55, 56^. Efforts must be made to develop such initiatives, probably by relying on new technologies such as smartphones and dedicated applications^55, 57^. Citizen science cannot, and must not, replace standard scientific practices^58^; they are complementary approaches with different strengths and limitations. However, citizen science could substantially contribute to our knowledge of biodiversity, especially if adapted programmes devoted to neglected taxa are highlighted^24^.

Considering the whole of biodiversity, and not only charismatic organisms, is a prerequisite for the development of efficient conservation plans, of prolific bioprospecting activities, and for enhancing our understanding of biodiversity on a global scale^8, 17, 59^. Many international projects have been developed since the Convention on Biological Diversity, illustrating an increased awareness of the astonishing diversity of functions and services that biodiversity supports^2, 3^. Nevertheless, while biodiversity declines at an unprecedented rate^60^, taxonomic bias is still a burden on biodiversity studies. It is urgent that we get rid of this burden and that we start embracing the whole of biodiversity.

## Methods

### Dataset

We downloaded all available occurrence records from the GBIF data portal in June 2016 (http://doi.org/10.15468/dl.hqesx6). 649 million occurrences were saved as a Darwin Core archive. Occurrences from this archive were extracted and imported into a SQL database, where data were indexed to reduce the computation time of subsequent queries. We focused on 24 taxonomic classes out of the 297 referenced in GBIF, excluding classes with less than 1 million occurrences (9.4 million occurrences from 19,000 species, had no class affiliation). We ended up with 626 million occurrences (NB_occ_) and 1.01 million species, representing more than 96% of the total number of occurrences and 84% of the total number of species in GBIF. All statistics were computed from this dataset.

### Taxonomic errors: imprecision and bias

For each class, we quantified the level of *taxonomic precision* as the proportion of occurrences with information at the species level or lower. We assessed *taxonomic bias* by computing and comparing the following statistics for each class: the total number of occurrences (nb_occ_), the median number of occurrences per species (med_/sp_) and the median absolute deviation, the proportion of species with at least one occurrence (p_>1_ = n_>1_/N), and the proportion of species with at least 20 occurrences (p_>20_ = n_>20_/N), where n_>i_ is the number of species with at least i occurrences and N is the number of known species for a given class. N was obtained using the GBIF taxonomic backbone (accessible at: http://doi.org/10.15468/39omei), by counting the number of distinct species with either the ‘accepted’ or ‘doubtful’ taxonomic status. This method excluded synonyms. Furthermore, we computed p_>20d_, the proportion of species with at least 20 spatially distinct occurrences. Two occurrences were considered spatially distinct when, using a global grid of 10*10 km cells based on the pseudocylindrical equal-area map projection Eckert IV, they fell in two different cells. We chose a threshold of 20 spatially distinct occurrences because it is a common threshold in niche modelling analyses^61^. Occurrences without spatial coordinates were excluded when computing the number of spatially distinct occurrences. We calculated how each class deviates from an ‘ideal’ sampling I, where each class is sampled proportionally to its number of known species (N). I = NB_occ_*(N/N_tot_) where N_tot_ is the total number of known species. To investigate the evolution of taxonomic bias over time, we excluded i) occurrences without a collection year and ii) occurrences recorded during the last 10 years because of the lag between recording and integration in the GBIF database (S. Gaiji, pers. comm.). The ‘ideal’ sampling I was calculated every ten years between 1956–2006 and deviations from these ‘ideal’ samplings were plotted for each class.

Statistics were computed at the ordinal level for Agaricomycetes, Amphibia, Aves, Insecta, Lecanoromycetes, Magnoliopsida, Mammalia and Reptilia using the same methods. These classes were chosen due to their relatively high number of occurrences and/or species, and because of the diversity of patterns they exhibited in our preliminary results. We also tried to cover a large taxonomic range (Tetrapods, Arthropoda, Plantae, Fungi) to include as much biodiversity as possible.

### Explanatory variables computed from the GBIF dataset

*Data origin*. In GBIF, the origin of an occurrence can be specified using a controlled vocabulary in the ‘basisOfRecord’ field. We delimited three categories, depending on whether recorded occurrences refer to a specimen (or object), an observation, or was of unknown origin. The “specimen” category (O_spec_) contained: fossil specimens, living specimens, material samples and preserved specimens. The “observation” category (O_obs_) consisted of: human observations, machine observations, unclassified observation and literature. The third category corresponded to the “unknown” option (O_unk_).

#### Date and Locality precision (Data completeness)

For each class, the proportion of temporal (p_-time_) and spatial inaccuracies (p_-space_) was computed as follows: p_-time_ = O_-time_/nb_occ_ and p_-space_ = O_-space_/nb_occ_, where O_-time_ is the number of occurrences lacking information regarding either the month, year or both, and O_-space_ is the number of occurrences missing coordinates or flagged as having geospatial issues in GBIF.

### External explanatory variables

#### Taxonomic research and societal preferences

Taxonomic research was quantified through the number of publications. We searched the Web of Science portal (apps.webofknowledge.com) with the following query for each order: “taxonomy” AND (“[order name]” OR “[family names]”), over the 1900–2016 period. The number of systematists, who are the producers of primary biodiversity data, would have been a better indicator but this could not be obtained due to the current architecture of Web of Science. We therefore used the publication metrics for taxonomic research from Web of Science as done previously^37^.

Public interest for a given species was estimated through the number of web page results, a proxy that has been proven to be reliable^22^. These numbers were obtained from Bing searches using the exact Latin name (e.g. “*Corvus corax*”) or a combination of the Latin name and the keyword “species” (e.g. “*Corvus corax*” + species). Bing and Google searches yielded similar results for the 4,000 species tested with both search engines (Supplementary Fig. 3), but only Bing allowed us to carry out a high number of searches. For each class, these searches were performed on the 1,000 species with the most occurrences (except for Pinopsida, which only had 902 species recorded in the GBIF) and then on a further 1,000 randomly chosen species. Each search was run twice to check for consistency.

### Statistical analyses

We favored medians (m) over means because of their robustness to outliers. For the same reason, we used the median absolute deviation (mad), which represents the median of the absolute deviation from the median, as a measure of statistical dispersion. In all analyses needing spatial or temporal information, O_-space_ and O_-time_ occurrences were ignored, respectively.

The relationship between data origin, completeness and year of record was investigated using multiple correspondence analyses (MCA). Analyses were done on three samples of five million random occurrences from our dataset. The variables were: class (24 categories), year of the record (categories: ‘<1900’, ‘1900–1949’, ‘1950–1974’, ‘1975–1999’, ‘2000–2004’, ‘2005–2009’, ‘2010–2014’, ‘> = 2015’), data origin (categories: specimen, observation, unknown), data completeness (categories: no problem, missing temporal information, missing spatial information, missing both). Because results can be hard to interpret when categories with very few observations are used^59^, each analysis was performed a second time ventilating the categories represented in less than 0.5% of the dataset.

To explore the relative impact of public interest and taxonomic research quantity on taxonomic bias, we used generalized linear models^62, 63^ (GLM). For each of the 24 classes, we looked at the effect of these two variables and their interaction on the number of occurrences per species in GBIF. We used an identical model for all classes, which was fitted using a negative binomial distribution to take into account overdispersion. Half of the GLMs were computed using the 1,000 best-represented species in GBIF (Best), while the other half used 1,000 random species referenced in GBIF (Random). Only one GLM was computed for Pinopsida because they had less than 1,000 species. Initial models were strongly influenced by extreme values and had poor resolution. Therefore we excluded outliers, which were identified when the number of occurrences or web search results was >Q_3_ + 4 * IQR, where Q_3_ is the third quartile value and IQR is the interquartile range. For each GLM, we checked the validity of the model by plotting the values of residuals against predicted values to test the homogeneity of residuals.

We performed all analyses using the R statistical software version 3.3.2 (https://www.R-project.org) with associated packages: FactoMineR^64^, ggplot2^65^, gridExtra^66^, MASS^67^, plyr^68^ and scales^69^.

## Electronic supplementary material

Supplementary information
