# Supplementary material for: Taxonomic bias in biodiversity data and societal preferences
Source: Sci Rep. 2017 Aug 22;7:9132. doi: 10.1038/s41598-017-09084-6 (PMC5567328; doi:10.1038/s41598-017-09084-6)
Supplement: Supplementary file 1 — Supplementary information [file 41598_2017_9084_MOESM1_ESM.pdf]

Taxonomic bias in biodiversity data and societal preferences

Supplementary material

Julien Troudet[1*], Philippe Grandcolas[1], Amandine Blin[2], Régine Vignes-Lebbe[1§], Frédéric Legendre[1§]

[1]Institut de Systématique, Evolution, Biodiversité, ISYEB – UMR 7205 MNHN CNRS UPMC EPHE, Sorbonne Universités, 45 rue Buffon 75005 Paris, France

[2]Outils et Méthodes de la Systématique Intégrative, OMSI – UMS 2700, MNHN CNRS, CP26, 57 rue Cuvier 75231 Paris Cedex 05, France

* corresponding author: julien.troudet@mnhn.fr

[§] These authors jointly supervised this work

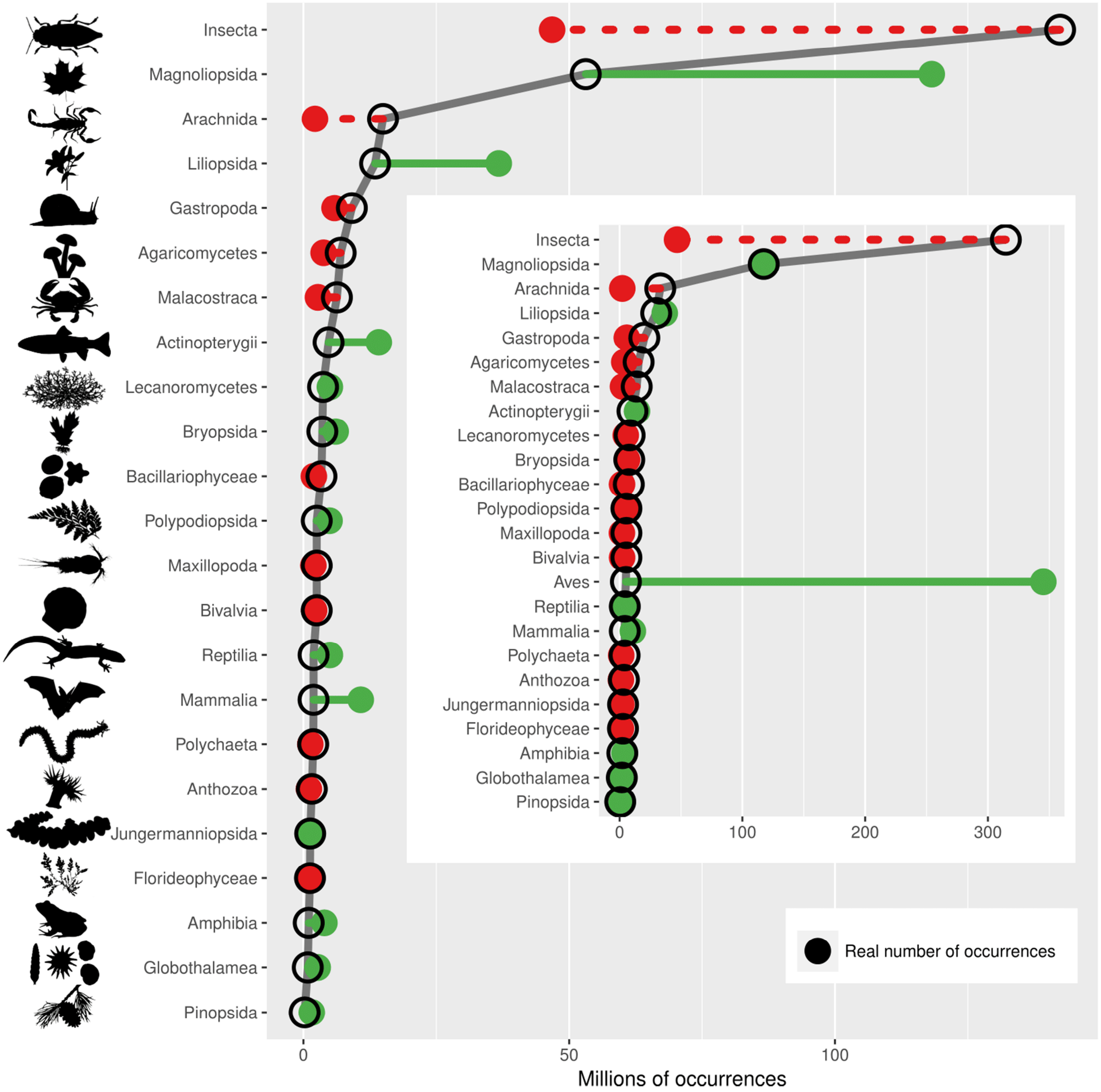

Figure S1: Taxonomic bias in biodiversity data occurrences. The grey line and black circles represent the 'ideal' number of occurrences per class, wherein each class is sampled proportionally to its number of known species. Green and red symbols show classes that are over- and under-represented in GBIF mediated data with regard to this 'ideal' sampling, respectively. The green and red dots represent the real number of data in the GBIF. The insert also depicts the taxonomic bias but includes Aves, the most over-represented class, in the calculation.

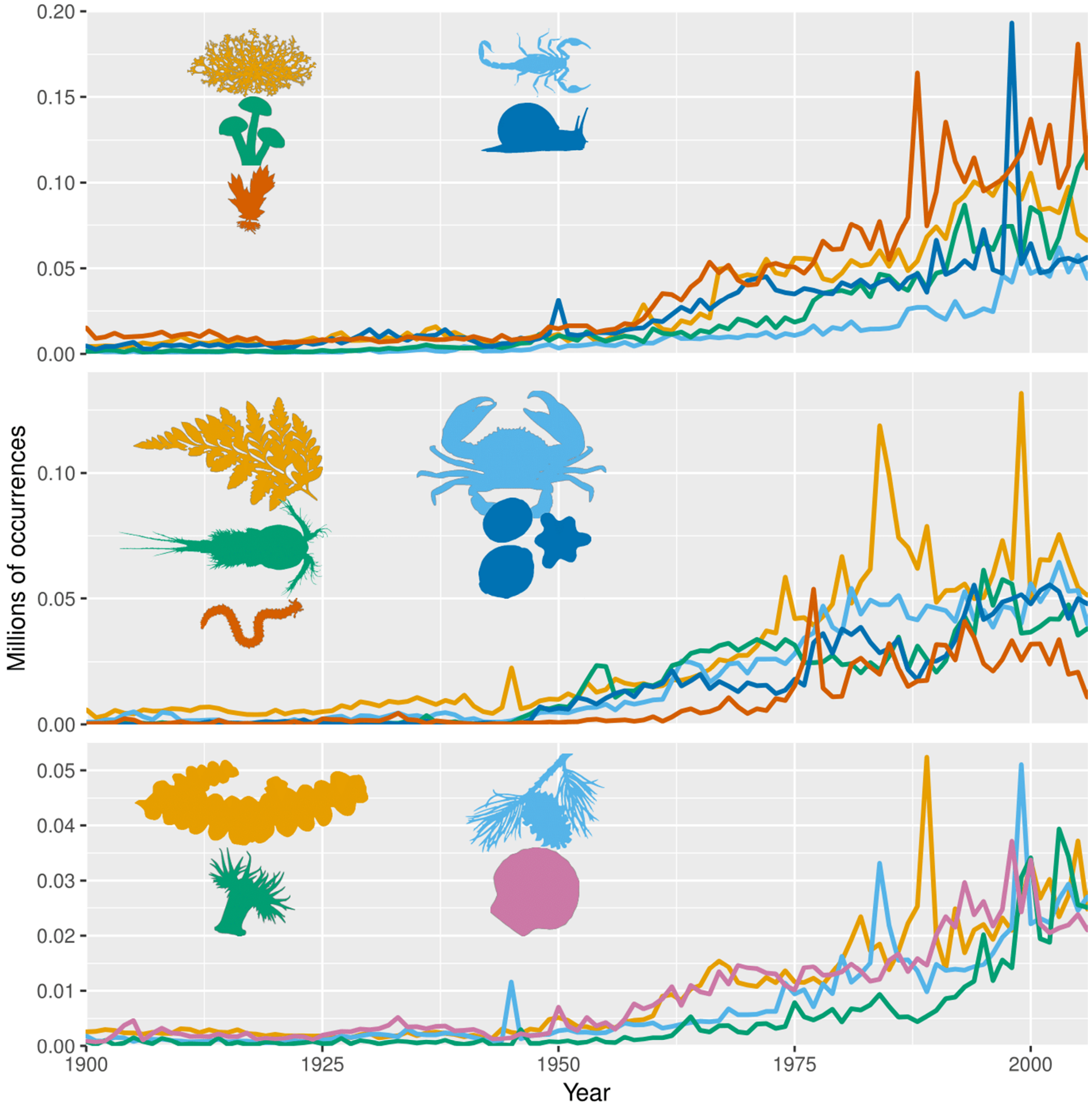

Figure S2. **Biodiversity occurrences recorded in the GBIF between 1900 and 2006**. For each curve, the number of occurrences is displayed year by year. *Top*: yellow = Lecanoromycetes; light blue = Arachnida; green = Agaricomycetes; dark blue = Gastropoda; orange = Bryopsida; *Middle*: yellow = Florideophyceae; light blue = Malacostraca; green = Maxillopoda; dark blue = Bacillariophyceae; orange = Polychaeta; *Bottom*: yellow = Jungermanniopsida; light blue = Pinopsida; green = Anthozoa; purple = Bivalvia.

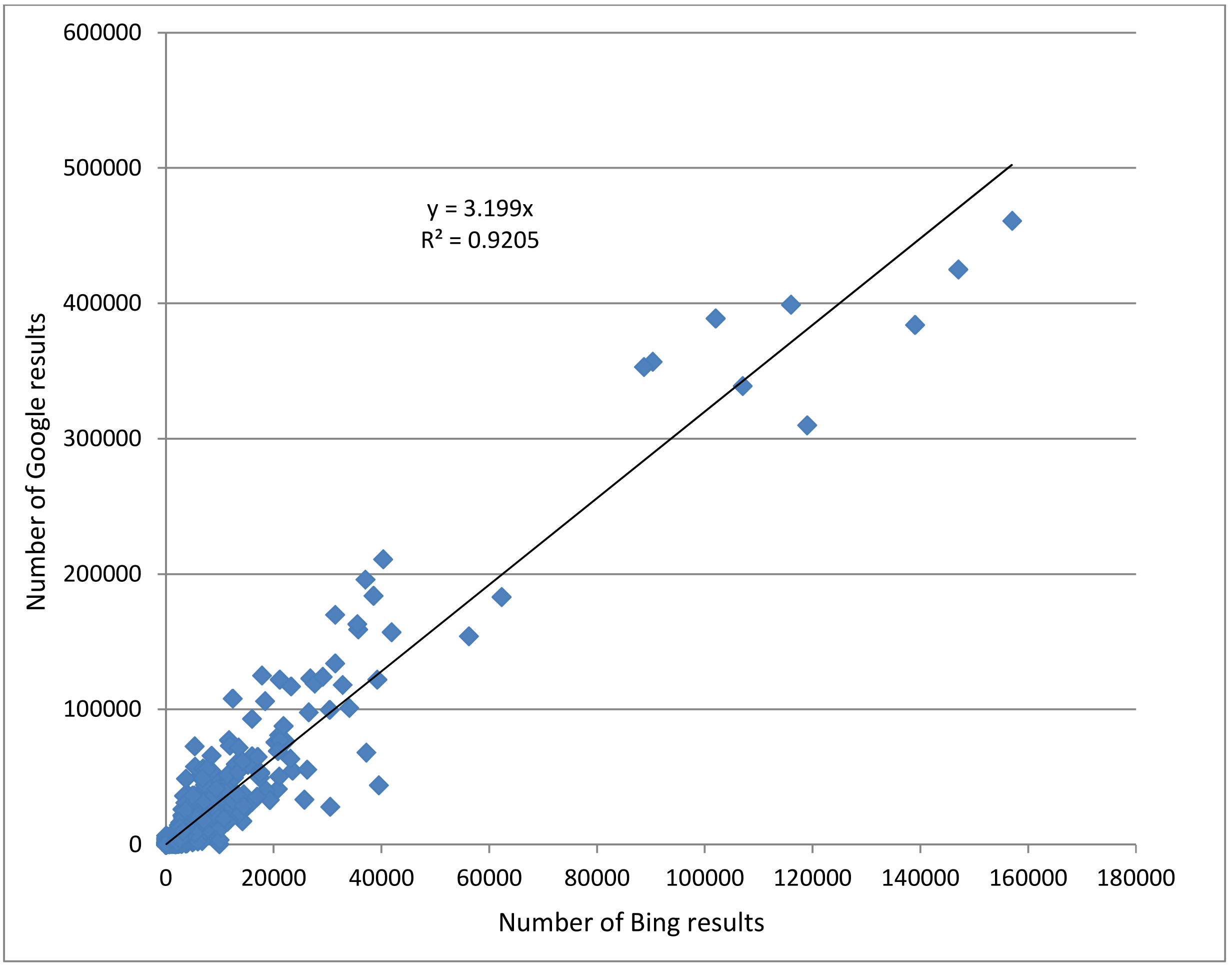

Figure S3. Relation between the number of Google search results and Bing search results for 4000 random species. We compared the number of web search results for two popular search engines. Using 4000 species from four classes (Aves, Magnoliopsida, Insecta and Liliopsida – 1000 species each), we found that the two search engines gave comparable results, with Bing returning fewer results than Google in general. Using Google for more requests was impossible because the script used was detected as potential spam.

Table S1. Positive relation between public interest and the number of occurrences for most classes. Results of the 47 negative-binomial GLMs assessing research and public interest influences on the amount of biodiversity data per class. Half of the GLMs were computed using the 1000 best-represented species in the GBIF (Best), while the other half used 1000 random species referenced in the GBIF (Random). Only one GLM was computed for Pinopsida because it had less than 1000 species. Influence cells filled with green are significant at a 5 % threshold. A text in blue indicates a positive influence while a text in red indicates a negative influence. Nb species = the number of species used in the GLM after removing outliers; pval = p-values; NA = not available (because no order information and therefore no research quantity was available for Pinopsida).

Class	Selected species	Nb species	Research influence	Public interest influence	Interaction influence	Research influence pval	Public interest influence pval	Interaction influence pval
Actinopterygii	Best	930	1.36E-06	7.08E-05	-1.71E-09	0.78	0	0.023
	Random	883	2.74E-05	8.41E-04	-1.93E-08	0.004	0	0.014
Agaricomycetes	Best	951	-8.74E-05	1.17E-04	1.54E-08	0.002	0	0.055
	Random	738	-1.40E-04	3.60E-02	1.47E-06	0.032	0	0.659
Amphibia	Best	916	-9.09E-05	4.31E-05	1.52E-08	0	0.573	0.058
	Random	875	1.14E-04	6.34E-04	-1.33E-08	0.024	0.076	0.714
Anthozoa	Best	910	-6.64E-05	4.60E-05	1.33E-07	0.273	0.304	0
	Random	744	-1.98E-04	2.08E-02	6.66E-06	0.101	0.002	0.198
Arachnida	Best	930	-2.81E-05	-5.22E-05	2.54E-08	0.021	0.376	0
	Random	799	-1.10E-05	1.71E-02	-4.69E-07	0.624	0.029	0.632
Aves	Best	930	-2.81E-05	-5.22E-05	2.54E-08	0.021	0.376	0
	Random	850	1.56E-05	1.22E-03	6.77E-09	0.182	0	0.277
Bacillariophyceae	Best	885	-5.96E-04	3.86E-04	1.28E-06	0.616	0	0.174
	Random	780	-3.46E-03	5.74E-02	-1.36E-04	0.011	0	0.23
Bivalvia	Best	928	-4.11E-05	1.20E-04	1.77E-08	0.082	0	0.16
	Random	755	5.41E-05	5.64E-02	-4.99E-06	0.313	0	0.087
Bryopsida	Best	905	1.21E-03	1.13E-03	-4.94E-07	0	0	0.079
	Random	846	4.16E-04	8.02E-02	-1.46E-05	0.366	0	0.672
Florideophyceae	Best	904	-1.98E-04	3.75E-04	6.82E-07	0.07	0	0
	Random	818	7.30E-04	4.95E-02	5.39E-06	0.002	0	0.665
Gastropoda	Best	718	3.54E-05	1.94E-05	2.45E-08	0.183	0.683	0.045
	Random	521	9.78E-05	7.85E-03	-3.32E-07	0.11	0.033	0.738
Globothalamea	Best	886	1.00E-03	1.43E-03	-4.39E-07	0	0.005	0.599
	Random	793	-4.77E-04	3.86E-02	4.86E-05	0.31	0.015	0.106
Insecta	Best	967	-1.95E-06	5.31E-05	-3.76E-10	0.246	0	0.216
	Random	769	5.04E-06	3.46E-02	-1.39E-07	0.369	0.013	0.601
Jungermanniopsida	Best	905	2.56E-04	1.80E-03	1.30E-06	0.405	0	0.013
	Random	850	7.74E-07	6.81E-02	2.66E-05	0.999	0.001	0.558

Lecanoromycetes	Best	961	-3.52E-05	6.68E-04	-8.86E-09	0.667	0	0.851
	Random	804	-9.23E-05	6.05E-02	6.27E-06	0.584	0	0.56
Liliopsida	Best	931	1.22E-05	9.30E-05	-1.19E-09	0.06	0	0.168
	Random	856	3.68E-05	9.23E-04	9.13E-09	0	0	0.615
Magnoliopsida	Best	959	2.76E-05	6.86E-05	-1.19E-09	0.003	0	0.205
	Random	768	-1.80E-05	3.34E-03	1.69E-08	0.17	0.001	0.863
Malacostraca	Best	906	-2.00E-05	2.45E-04	-1.23E-08	0.002	0	0.001
	Random	757	-1.61E-05	8.82E-03	1.09E-06	0.392	0.156	0.154
Mammalia	Best	913	8.80E-06	1.05E-04	-1.96E-09	0.024	0	0
	Random	800	1.36E-05	8.96E-04	-6.35E-09	0.049	0	0.1
Maxillopoda	Best	889	1.54E-04	3.99E-04	-2.80E-08	0.017	0	0.54
	Random	835	-1.24E-05	1.85E-02	3.38E-06	0.898	0.012	0.51
Pinopsida		796	NA	3.22E-04	NA	NA	0	NA
Polychaeta	Best	790	-2.27E-04	8.01E-04	1.59E-07	0.053	0	0.389
	Random	712	-3.83E-04	3.73E-02	9.18E-06	0.212	0.01	0.519
Polypodiopsida	Best	938	-8.59E-05	8.04E-05	1.62E-08	0.174	0	0.335
	Random	785	4.95E-04	5.72E-02	-6.84E-06	0.048	0	0.473
Reptilia	Best	940	8.99E-06	5.12E-05	3.12E-09	0.627	0.18	0.19
	Random	794	4.01E-05	4.62E-04	1.09E-08	0.104	0.04	0.448
